# Hemolysis and von Willebrand factor degradation in mechanical shuttle shear flow tester

**DOI:** 10.1007/s10047-020-01219-3

**Published:** 2021-02-09

**Authors:** Yasuyuki Shiraishi, Yuma Tachizaki, Yusuke Inoue, Masaki Hayakawa, Akihiro Yamada, Michinori Kayashima, Masanori Matsumoto, Hisanori Horiuchi, Tomoyuki Yambe

**Affiliations:** 1 Department of Preclinical Evaluation, Pre-Clinical Research Center, Institute of Development, Aging and Cancer, Tohoku University, Sendai, Japan; 2 Department of Medical Engineering and Cardiology, Pre-Clinical Research Center, Institute of Development, Aging and Cancer, Tohoku University, 4-1 Seiryo-machi, Aoba-ku, Sendai, 980-8575 Japan; 3 Department of Blood Transfusion Medicine, Nara Medical University, Nara, Japan; 4 Department of Molecular and Cellular Biology, Institute of Development, Aging and Cancer, Tohoku University, Sendai, Japan

**Keywords:** Shuttle shear flow tester, Hemolysis, Von Willebrand factor, Large multimer

## Abstract

Chronic blood trauma caused by the shear stresses generated by mechanical circulatory support (MCS) systems is one of the major concerns to be considered during the development of ventricular assist devices. Large multimers with high-molecular-weight von Willebrand factor (VWF) are extended by the fluid forces in a shear flow and are cleaved by ADAMTS13. Since the mechanical revolving motions in artificial MCSs induce cleavage in large VWF multimers, nonsurgical bleeding associated with the MCS is likely to occur after mechanical hemodynamic support. In this study, the shear stress (~ 600 Pa) and exposure time related to hemolysis and VWF degradation were investigated using a newly designed mechanical shuttle shear flow tester. The device consisted of a pair of cylinders facing the test section of a small-sized pipe; both the cylinders were connected to composite mechanical heads with a sliding-sleeve structure for axial separation during the withdrawing motion. The influence of exposure time, in terms of the number of stress cycles, on hemolysis and VWF degradation was confirmed using fresh goat blood, and the differences in the rates of dissipation of the multimers were established. The plasma-free hemoglobin levels showed a logarithmic increase corresponding to the number of cycles, and the dissipation of large VWF multimers occurred within a few seconds under high shear stress flow conditions.

## Introduction

Chronic blood trauma caused by shear stresses generated by mechanical circulatory support (MCS) systems is one of the major concerns to be considered during the development of ventricular assist devices. The shear stresses induced by the designs of the MCSs or cannulations are anticipated to be a critical cause of nonsurgical and nonphysiological lysis of blood and degradation of the von Willebrand factor (VWF) [1–3]. Large multimers with high-molecular-weight VWF are extended by the fluid forces in a shear flow and are cleaved by ADAMTS13 (a disintegrin and metalloproteinase with a thrombospondin type 1 motif, member 13) under high shear stress flow conditions in the cardiovascular system [4–8]. Previously, empirically estimated hemolysis or VWF degradation models were developed to predict the concentration and accumulation of shear flows in MCSs through their development processes [9–12].

Extant studies on the hemolysis and cleavage of the VWF associated with increasing shear flow were based on in vitro tests that used Couette-type blood shearing devices to determine empirical constants, including the thresholds of blood trauma activation [4, 13, 14]. However, experimental analyses using steady flows neglected the time-varying effects and the complicated properties of natural blood flow. To date, wall shear characteristics and their relationship to blood trauma are poorly understood because of the complexity of interaction between the erythrocytes and plasma at the boundary walls.

The dynamics of the VWF elongational flow corresponding to hemorrhage are associated with the shear flow. The present study provides an experimental analysis of hemolysis and the VWF degradation degree for goat blood using a newly designed shuttle shear flow tester. The maximal velocity in the tester was designed according to the threshold Reynolds stress that would cause hemolysis, i.e., 400–800 Pa, as described in previous studies [10, 15]. The present study aimed to examine the lysis of red blood cells and VWF degradation in the shear stress flow range of approximately 10–600 Pa under the assumption of the threshold of either the VWF degradation or the hemolysis with time-dependent variations in blood flow velocity.

## Materials and methods

### Design of shear flow tester

**Fig. 1:**
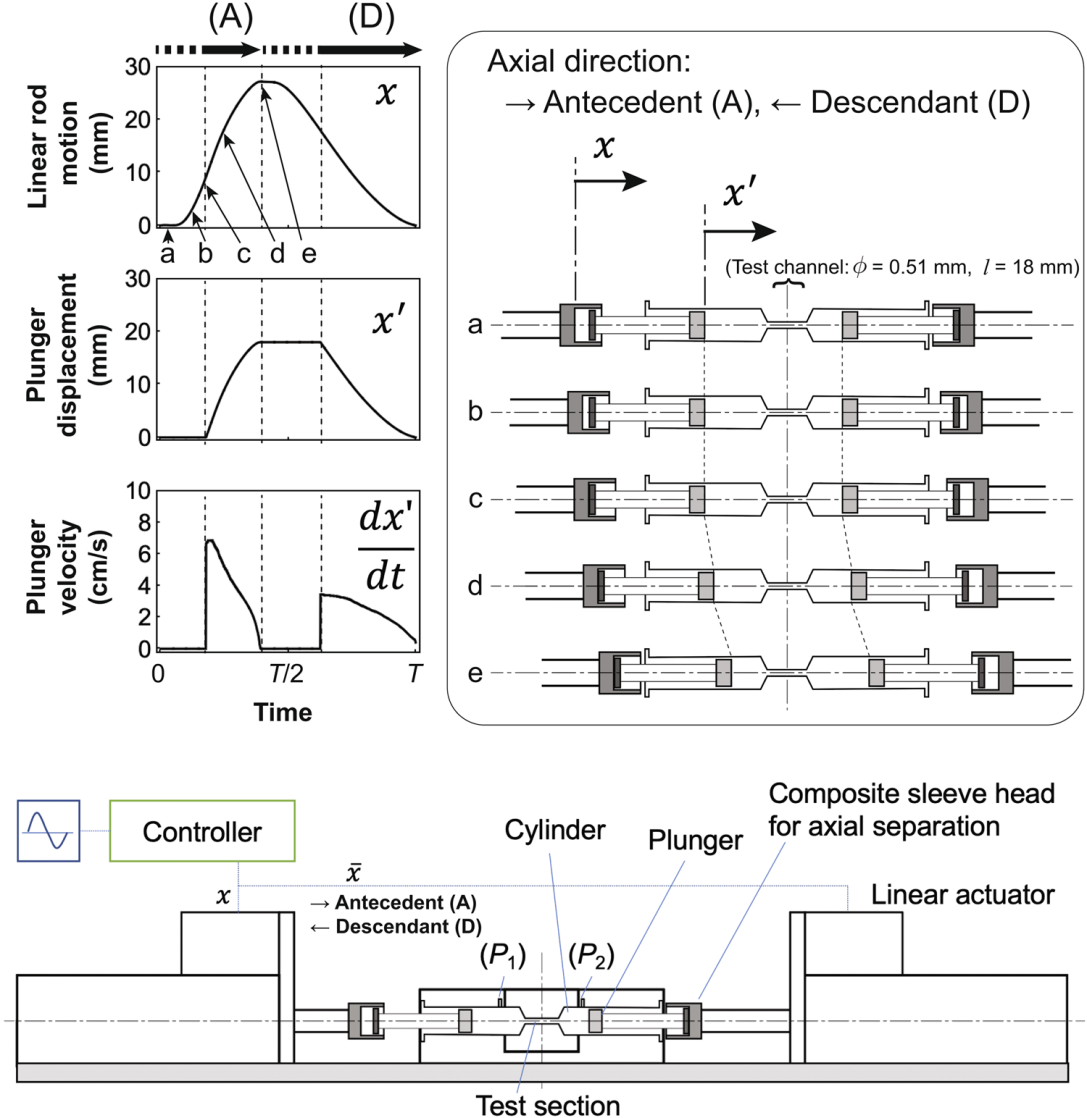
Schematic illustrations of shuttle shear flow tester (bottom) with waveform sequences for linear actuator rod motion, plunger displacement, and plunger velocity (top left), and mechanical sequence of composite mechanical head with sliding-sleeve structure for axial separation (top right)

We designed a new bench-top pulsatile reciprocating-type blood shear tester. Figure 1 shows the overview of the system, which is composed of the following three sections: (a) two linear actuator controllers (PWA-M6H010R, Oriental Motor, Tokyo, Japan), (b) tandemly coupled cylindrical connectors with syringes, and (c) a flow test section consisting of a thin pipe with a diameter of 0.51 mm and length of 18 mm.

The outer housing of each syringe was placed on a holder fixed to the test bench. The test section with the thin pipe was connected between the two syringes. The two linear actuators were driven by a programmable pulse generator, and each was actuated in the reverse phase to achieve the required relative displacement. To design a microprocessor control system that could drive the relative motion between the two actuators facing each other, cross-wiring was used for the drive pulse signal. The articulation between the linear actuator rod and syringe plunger was tandemly coupled using a connector with a composite mechanical head and a sliding-sleeve structure for axial separation during the withdrawing motion (Fig. 1). The reciprocation of the shuttle shear tester was designed with a couple of actuators, and each displacement and velocity of the cylinder motion was basically controlled independently. In this study, we arranged the motion of the two cylinders synchronously by placing the normal and the reverse inputs to each controller to achieve parallel motion of a couple of plungers. The waveforms of the changes in displacement of cylinders were designed for smooth motion so that there is no sudden change in motion velocity, on the other hand, the distribution of the velocity values is similar to the arcsine distribution in which the high-speed duration is limited due to the time-varying cylinder movement. By making the cylinder motion as a fast push-and-slow pull, we primarily intended to widen the range of plunger velocity variations by the different maximum velocity setting at the push and the pull phases of each cylinder. Then, the antecedent separation phase was set as a mechanically imperceptive period of displacement at the beginning of the active pressurizing phases (Fig. 1, phases through ‘b’–‘d’). Both plunger-ends stopped at the position “e”, and they had no contact the end of the syringe sleeve directly to prevent red blood cells from being smashed. The resolution of the actuator was 0.01 mm, and the minimum control volume generated by the plunger was 0.34 µL. As the actuator generated an afterload-independent motion, the displacement distance of the plunger was linearly dependent on the waveform input.

### Shear stress test using blood

The velocity of the reciprocating movement for each direction was set and driven separately by the linear actuator controller. The maximal velocities of the linear rods in the two directions were set to $6.8 \times 10^{ - 2}$ and $3.4 \times 10^{ - 2}$ m/s. This mechanism was provided to apply hemolytic shear above 600 Pa in one of the directed paths and to force VWF degradation shear in both directions through the test channel.

Blood was drawn from an adult healthy Saanen goat prior to the measurements. The blood samples were anticoagulated by a CPDA-1 (citrate phosphate dextrose adenine) solution (Karmi-CA, Kawasumi Laboratories, Tokyo, Japan). The measurements were performed three times using the same blood from a goat. All institutional and national guidelines for the care and use of laboratory animals were observed, and all the protocols were approved by the appropriate institutional committees.

A pair of syringes was filled with the blood using the connectors and firmly placed on the test bench. The total volume of the blood in the test circuit was 2 mL. Shear tests were performed thrice under the same driving conditions. The tests were performed at room temperature (25 ± 2 °C). The whole blood samples were collected at 1, 2, 5, 10, 25, and 50 stress cycles by the tester in each repetition. The cyclic frequency for the reciprocating motion was 0.5 Hz. The maximal velocities of the blood forced in directions A and B (Fig. 1) were 11.7 and 5.9 m/s, respectively. The upstream and downstream pressures in the test channel, which were used to calculate the differential pressure, were measured using pressure transducers (Meritrans DTXplus, Merit Medical Japan, Tokyo, Japan) and a dynamic strain amplifier (DPM-6H, Kyowa Electronics Instruments, Tokyo, Japan). The pressure was digitized and recorded (PowerLab; LabChart 8, ADInstruments, Inc., Dunedin, New Zealand) for analysis. The shear stress *τ* was calculated using the differential pressure and test channel specifications as follows [16]:

1 $$\tau = \frac{{P_{1} - P_{2} }}{4}\frac{d}{l},$$

where $P_{1}$ and $P_{2}$ are the upstream and downstream pressures, respectively, and $d$ and $l$ are the diameter and length of the test channel, respectively. The hematocrit levels were measured in each session.

### Laboratory assays

After the stress test, the blood samples were collected in sodium-EDTA and centrifuged to provide the plasma. The changes in the plasma-free hemoglobin level were measured for each sample using a spectrophotometer (SP-300, Optima, Tokyo, Japan). Absorbance measurements were performed using the spectrophotometer that was previously calibrated with control hemoglobin (Hemoglobin B test kit, (Fujifilm Wako Pure Chemical Corp., Osaka, Japan) by a method that used the sodium lauryl sulfate reagent (Sulfolyser, Sysmex, Kobe, Japan) at a wavelength of 540 nm [17]. A percentage change in the delta plasma-free hemoglobin level (ΔpfHb) was considered to be the difference between the control plasma-free hemoglobin level (pfHb) and ΔpfHb level [18]. The analysis and handling of the blood were carried out according to ASTM F1841-97 (2017) recommendations where applicable.

VWF degradation was examined under the cyclic conditions and compared with the changes in hemolysis. We performed VWF multimer analysis based on Ruggeri and Zimmerman’s methods [19]. The loss of large multimers was examined using the scanned blotting results obtained from the VWF multimer analysis. Each Western blot probing VWF was visualized by the chemiluminescence ImmunoStar Zeta kit (Fujifilm Wako Pure Chemical Corp., Osaka, Japan) using a biomolecular imager (ImageQuant LAS4000, GE Healthcare, Chicago, IL, USA), as described in previous studies [2, 7]. The large multimer index was densitometrically defined by the integrated values of the large multimer areas normalized by the total VWF multimers, as described in the literature [7]. The large multimers were classified and defined according to the weight bands that were larger than the tenth molecular weight band from the weight bands of low-molecular-weight VWFs. The integrated values of the large multimer band spectral areas were normalized by the total VWF multimers. The index was obtained as a percentage of the ratio of the measured large multimers against the ratio of the large multimers of the control blood sample (pretest sample) in the same set of Western blot lanes. The index was calculated by performing densitometric analysis using ImageJ (National Institute of Health, Bethesda, MD, USA) and Mathematica (version 11, Wolfram Research, Champaign, IL, USA).

### Statistical analysis

Continuous variables were presented as mean ± SD, and a *p* value of less than 0.05 was considered to be statistically significant. Correlations between variables were assessed using Pearson’s correlation test. The Mann–Whitney *U* test was used to calculate significant differences between the groups. All the statistical analyses were conducted using Stata (version 15, StataCorp, College Station, TX, USA).

## Results

### Changes in shear stress applied to blood in tester

**Fig. 2:**
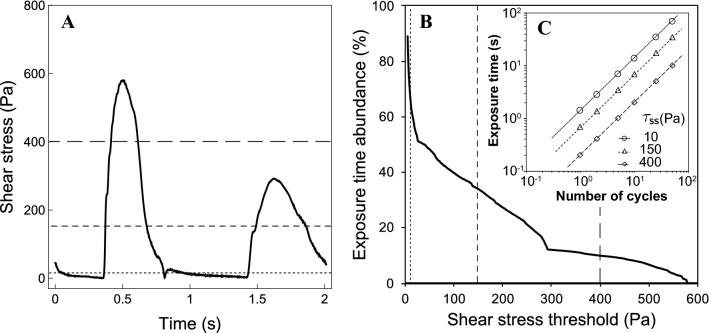
Shear stress waveform obtained from differential pressures measured at upstream and downstream ports in shear flow tester (left, **a**), and relationships between exposure time abundance (right, **b**), and shear stress threshold and between exposure time and number of cycles at stress exposure levels of 10, 150, and 400 Pa (right, **c**)

Figure 2a shows the change in the shear stress obtained from the differential pressure according to Eq. (1). The maximal shear stress in the antecedent (A) and descendant (D) directions was 580 and 292 Pa, respectively. These maximal values were obtained at the beginning of each motion during the forward or backward motions of the linear actuator rods. Figure 2b shows the change in the exposure times against the shear stress threshold in one cycle. The exposure time abundance was calculated as the integrated exposure time above the threshold. Since both antecedent and descendant directory paths were present in one cycle, the relationship between the exposure time abundance and shear stress exhibited different slopes at a threshold of around 300 Pa, and the exposure time abundance and shear stress showed negative correlations (Fig. 2b). The relationships between the total exposure times and the number of cycles over the thresholds at 10, 150, and 400 Pa are presented as examples in Fig. 2c; here, the exposure times were 68.2% (1.4 s), 34.0% (0.7 s), and 10.0% (0.2 s) in one cycle, respectively. The exposure times of paths (A) and (D) shown in Fig. 1 above these thresholds were obtained. The mean exposure time on path (A) against the total exposure time was 52.7 ± 13.6% below 300 Pa. The shear stress above 300 Pa was investigated only on path (A).

### Changes in hemolysis according to number of cycles

**Fig. 3:**
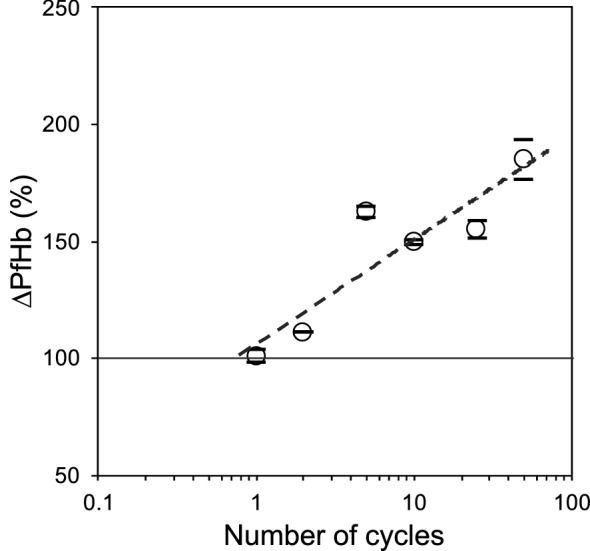
Changes in plasma-free hemoglobin levels obtained in tester with respect to number of cycles

The hematocrit level was 27.4 ± 0.6%, and no significant differences were found among the samples. ΔpfHb exhibited a linear increase with the logarithm of the number of cycles, as shown in Fig. 3 (*p* = 0.001, *N* = 3).

### Changes in large multimer index

**Fig. 4:**
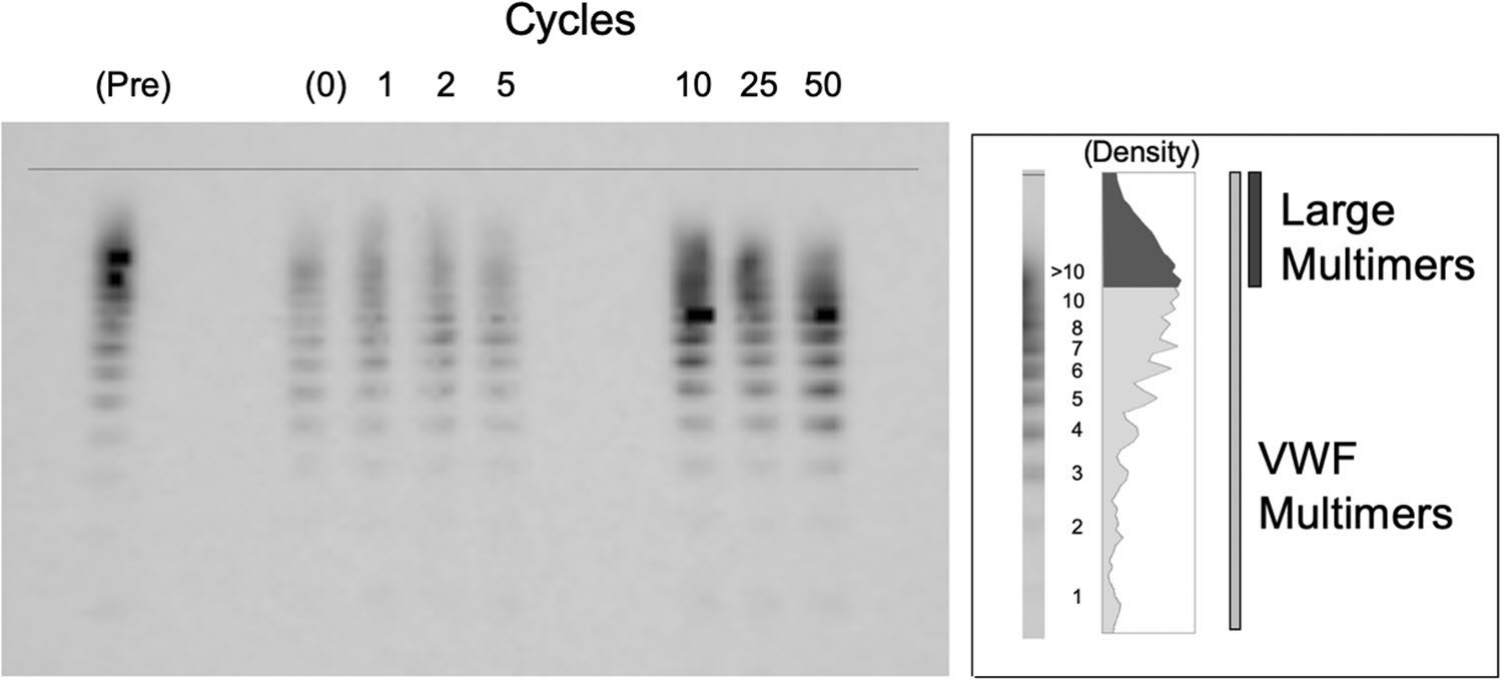
Series of lanes representing VWF multimers. “Pre” indicates the pretest, and the numbers at the top of the photograph indicate the number of cycles (left). Schematic illustration of densitometric analysis of bands with VWF and large multimers (right)

**Fig. 5:**
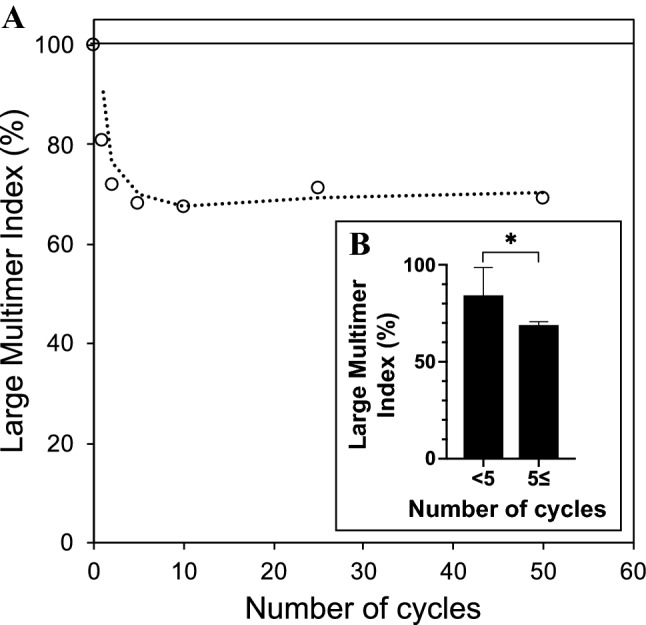
Relationship between large multimer index and number of cycles (**a**). The dotted line represents the moving average. Comparison of large multimer indices obtained by thresholds at five cycles (**b**, *p* = 0.03)

Figure 4 shows a lane profile obtained from the samples after application of cyclic shear stress. The relationship between the large multimer index and the number of cycles is shown in Fig. 5a. The transient dissipation corresponding to an increase in the number of cycles was investigated, and the large multimer indices were observed to decrease as the number of cycles increased. The large multimer indices corresponding to less than five cycles of shuttle stress were significantly larger than those corresponding to more than five cycles of shuttle stress (Fig. 5b, *p* = 0.03).

## Discussion

In this study, a shuttle shear flow tester with two reciprocating cylinders was developed. Hemolysis and VWF degradation were examined for different numbers of shuttle cycles as shear flow was produced under high shear stress conditions generated by MCSs. The feature of the proposed testing system was that it had a small-sized pipe (~ 0.5 mm) with a relatively long length (18 mm). The advantage of the tester was that it provided elevated stress implications under a laminar assumption with the pipe dimensions. Hence, the shear stress was estimated where the viscosity was constant. Although blood is a non-Newtonian fluid with shear thinning characteristics, it was treated as a Newtonian fluid in the present study because of the high shear rate (~ 10^5^ s^−1^).

The present study showed a larger increase in the plasma-free hemoglobin level for the maximum exposure time of 200 s (Fig. 3) than the scaled hemolysis study described in the literature [20]. The differences in hemolysis between the present study and previous study could be attributed to the fact that in the present study, the shear stress was induced by a reciprocating shuttle flow. Moreover, the effect of sudden onset of blood trauma was not tested in the present study. In addition, because the normal morphometry of goat erythrocytes is approximately two times smaller than that of human erythrocytes, the critical velocity of the shear stress exposure for goat blood cells was estimated to be higher than that for human blood cells [16, 21].

As previously reported, the blood trauma corresponding to nonsurgical bleeding was related to the continuous flow generated by MCSs [1, 2, 7, 9, 22, 23]. On the other hand, the clinical relevance of the revolving effects of MCSs on VWF fragmentation followed by a decrease in large VWF multimers has not yet been clarified [24, 25]. The continuous flows provided by MCSs exhibit high pulses under the constant revolving condition for circulatory support, which may cause a wide range of shear stresses during cardiovascular support [26]. The present study demonstrated the effect of the shuttle shear flow on the degradation of the large VWF multimers under varying shear stress. The significant decrease in large multimers was investigated for an exposure time of 6.8 s (5 cycles) at a shear stress above 10 Pa in 2-mL blood samples. Since there were no discernible differences in the large multimer degradations at different exposure times over the threshold, the large multimers were considered to be fully dissipated by the shear stresses generated by the tester. The whole volume of the test blood used in each condition was 2 mL, and the volume shearing in a reciprocating stroke was 0.63 mL (32%) of the test blood. By the increase of the number of cycles below the conditions of 5 cycles, the degradation of large multimers decreased as the number of cycles increased. We considered the degradation changes in the large multimer were associated with the integrated shear stress amplitude. The degradation changes in Fig. 5 indicated the saturation at the number of cycles had reached to 5 cycles, at which the large multimer index exhibited to be approximately 68.9 ± 1.7% (SD) that was. Therefore, the large multimer saturation ratio was related to the sheared volume. Although some sheared volumes were to be mixed with the residual volume in the test chamber with plungers, the mixed ratio could be low due to the low Reynolds numbers at the portions of the shear channel and of the plunger chamber that was to be laminar. Hence, the shear stress could be applied limitedly on the reciprocating stroke volume repeatedly.

Although the Reynolds stress of the small pipe was similar to the range observed in the artificial MCSs, the continuum assumption failed for the small size of the microcirculation channels. Moreover, special considerations need to be made with regard to the relationships between shear stress and degradation in the accelerated shuttle flow tester under the conditions of jet and local turbulence.

While hemolysis increased relatively corresponding to the exposure time, the VWF degradation exhibited faster dissipation within five cycles in the tester. This result suggested that the increase in the cyclic shear stress yielded an A2 contour elongation followed by cleavage by ADAMTS13 in a few seconds when the shear stress reached the thresholds. For the design of rotary-type MCSs, the shear stress above 400–800 Pa exhibited significant hemolysis in vitro [27]. Sharp et al*.* investigated the threshold at around 150 Pa in needles regardless of the exposure time [16]. Considering that the present results showed a logarithmic increase in hemolysis related to cyclic stress with exposure of the blood to shear stress higher than the threshold, the dissipation through hemolysis would occur by the alternating succession of mechanical damage to erythrocytes rather than a sudden onset of shear stresses. In addition, the results of the acute changes in the degradation of the large VWF multimers were consistent with those obtained in previous works; that is, the sensitive elongation of VWF bindings related to the force that acts as a long-lasting impediment to refolding would accelerate the rate of cleavage by ADAMTS13 [28].

Our findings showed that the degradation of large VWF multimers was caused by the accumulation of cyclic shear flow, which was followed by the dissipation of the large VWF multimers in the shuttle flow tester. Several studies reported unfolding states, large structural transitions, and changes in VWF conformation at high shear stresses of around 3–10 Pa [28–31]. The unfolding state is kept longer than 140 s under the high shear stress condition, as described in a previous study [28]. The exposure time abundance intermittently applied above 10 Pa in the tester was notably short. Therefore, VWF degradations occurred because of repeated shear stresses.

In this study, whole blood was used as a test medium. The results indicated the continuous increase of plasma-free hemoglobin during the cyclic tests. Interestingly, the changes in high-molecular-weight (HMW)-VWF showed a decrease and dissipation in the volume loaded in the system. Our findings suggest that the early fragmentation under the high shear stress condition remarkably increased in the hemolytic plasma. In the low Reynolds number fluid behavior, as the mixing dynamics with turbulence is impaired, the antigen and protease stay in place without disturbance in the test blood. On the other hand, the reaction of ADAMTS13 is active and progresses at the moving flow boundary interface by mixing even in the low Reynolds number. Bartoli et al. previously reported that ADAMTS13 functional activity was low when the concentration of plasma-free hemoglobin was high in the steady turbulent flow [32]. ADAMTS13 activity may be affected by the hemoglobin volume concentration discharged in plasma under the condition of the early stage of increasing release of hemoglobin from the red blood cell with high fragility. They speculated that ADAMTS13 antigen was impaired by hemoglobin, followed by possible thrombogenesis in the whole blood, but the hemoglobin concentration threshold is required for the changes in ADAMTS13 antigen on HMW-VWF multimers. Therefore, we need more substantial work to clear the relationship between the hemolysis and VWF in the shearing flow, including the analysis of the ADAMTS13 antigen or its activity under the stressed condition of the blood. In the future work, the variation of the stressed state using separated plasma or isolated red blood cell is to be investigated for more quantitative analysis to clear the threshold of HMW-VWF multimer impairment characterized by the different test conditions.

The threshold values of the shear stresses that cause blood trauma are valuable because these values can be used to prevent any increase in nonsurgical bleeding during the use of MCSs and to design blood-contacting devices operating in high blood velocity regions. The examination using the variable shear flow tester can provide the shear stress threshold and help in reducing the occurrence of mechanical stresses in MCSs and improving the condition of patients requiring mechanical circulatory support. The system represented the wide range of blood flow shear from physiologically low stress to supraphysiological rate with variable shearing periods that could simulate the aortic stenotic condition as well as the shear corresponding to the rotary blood pump mechanisms followed by acquired von Willebrand syndrome.

## Conclusions

In this study, the shear stress and exposure time related to hemolysis and VWF degradation were investigated by testing fresh goat blood in a newly designed shuttle shear flow tester. The influences of exposure time on hemolysis and VWF degradation were determined under the shuttle shear stress condition, and the differences in the rates of dissipation of the multimers were established. The plasma-free hemoglobin levels showed a logarithmic increase corresponding to the number of cycles, and the dissipation of large VWF multimers occurred within a few seconds under high shear stress flow conditions. Our results showed similar incremental changes in the degradation of VWF and the hemolysis existed in the overlap range under the supraphysiological shear stress condition. On the other hand, each threshold of shear stress associated with the activity of VWF and ADAMTS13 interactions as well as the erythrocyte membrane rupture remains to be seen in the laminar shuttle shear flow tester.

## Limitations

This study has several limitations. The blood sample was drawn from an animal, and the effects of individual differences on blood trauma were not verified. The characteristics of blood as a test material were examined in this study using whole blood. Since there is a significant change in the hematocrit levels in tubes with very small diameters, the obtained results may change for centrifugal blood pumps or MCSs used in natural cardiovascular systems. Moreover, as the plasma-free hemoglobin may activate leukocyte elastase, the VWF degradation may be induced by the proteinase other than ADAMTS13 in the limited test volume after the increase of hemolysis.

## Appendix

### Calculation of shear stress for test cylinder with plunger and thin-diameter pipe

The displacement of the plunger is 0.018 m, and the internal diameter of the cylinder is 0.0067 m. Hence, the total volume generated by a stroke is

A1 $$V_{{{\text{stroke}}}} = 6.3 \times 10^{ - 7} \,{\text{m}}^{{3}} .$$

Consider the residence times along the two direction paths A and D as $t_{{\text{A}}} = 0.430\; {\text{and}}\;t_{{\text{D}}} = 0.750$ s and the internal diameter as $d = 5.1 \times 10^{ - 4}$ m for the channel. Then, each mean flow rates $\overline{Q}_{{\text{A}}}$ and $\overline{Q}_{{\text{D}}}$ is calculated as

A2 $$\overline{Q}_{i} = \frac{{V_{{{\text{stroke}}}} }}{{t_{i} }},$$

where $i = {\text{A}}$ or ${\text{D}}$. Therefore, the mean velocities $\overline{U}_{{\text{A}}}$ and $\overline{U}_{{\text{D}}}$ are 7.1 and 4.1 m/s, respectively.

Then, the Reynolds numbers (${\text{Re}}_{{{\text{Amean}}}}$, ${\text{Re}}_{{{\text{Dmean}}}}$) calculated by Eq. (A3) at the mean flow velocity in the paths A and D are 1045 and 600, respectively:

A3 $${\text{Re}}_{{i{\text{mean}}}} = \frac{{\overline{U}_{i} \times d}}{\nu },$$

where *i* = A or D; viscosity of the blood, $\mu = 3.5 \times 10^{ - 3} \,{\text{N}}\,{\text{s/m}}^{{2}}$; $\nu = 3.5 \times 10^{ - 6} \,{\text{m}}^{{2}} {\text{/s}}$. The maximal plunger velocities in the paths A and D are 11.7 and 5.9 m/s, respectively; therefore, the peak Reynolds numbers (${\text{Re}}_{{{\text{Apeak}}}}$, ${\text{Re}}_{{{\text{Dpeak}}}}$) at each peak instantaneous flow are 1700 and 854, respectively.
